# A massive calcification and ossification of the transverse sinus and the neighbouring dura mimicking meningioma

**DOI:** 10.1186/1471-2377-13-143

**Published:** 2013-10-11

**Authors:** Zhiqin Xu, Changbao Su, Yu Xiao

**Affiliations:** 1 Department of Neurosurgery, Peking Union Medical College Hospital, Chinese Academy of Medical Sciences, No.1 Shuaifuyuan, Dongcheng district, Beijing 100730, P. R. China; 2 Department of Pathology, Peking Union Medical College Hospital, Chinese Academy of Medical Sciences, Beijing 100730, P. R. China

**Keywords:** Intracranial calcification, Intracranial ossification, Transverse sinus, Dura

## Abstract

**Background:**

Although small calcifications of the dura and the transverse sinus occur frequently, large, single intracranial calcifications originating from the transverse sinus and the neighbouring dura are rare.

**Case presentation:**

A 47-year-old man was admitted to the hospital for a right occipital headache that had persisted for two weeks. There was no neurological deficit. Normal skull X-ray and computed tomography (CT) scans revealed an irregular, calcified, intracranial lesion of approximately 4.4 × 4.0 × 2.5 cm in volume in the right occipital region. Via surgery, a bone-hard, poorly vascularised, pink mass originating from the right transverse sinus and the convex dura of the right cerebellar hemisphere, as well as the cerebellar tentorium, was completely removed. Pathological examination yielded a diagnosis of fibrous connective tissue with hyaline degeneration, calcification and ossification with no indication of neoplasia or inflammation.

**Conclusions:**

We report a rare case of massive calcification and ossification of the transverse sinus and the neighbouring dura mimicking meningioma. Degenerative calcification and ossification may serve as a rare differential diagnosis of diseases, such as meningiomas, in the transverse sinus and the neighbouring dura.

## Background

Intracranial calcification may occur physiologically or pathologically and may be observed in asymptomatic individuals. Clinical assessment and laboratory investigations are required to determine whether these changes are physiological, idiopathic, neoplastic, inflammatory, traumatic, caused by metabolic disease, or manifestations of a generalised disease, such as hyperparathyroidism, vitamin D toxicity, Aicardi-Goutières syndrome, cerebroretinal microangiopathy with calcifications, COL4A1-related disease, Degos disease, Krabbe disease, Alexander disease, mitochondrial disease, tetrasomy 15, Hutchinson-Gilford progeria syndrome or chronic renal failure [1-3].

Certain intracranial tumours, especially oligodendrogliomas and craniopharyngiomas, tend to undergo calcification. Tumour calcifications were observed in approximately 83% of craniopharyngiomas [4] and 40% of intracranial oligodendrogliomas [5]. Approximately 22 to 62% of benign meningiomas were reported to contain calcification, while nearly all malignant meningiomas lacked any calcification [6-8]. Metaplastic meningioma is a rare subtype of benign meningiomas, which encompass a broad range of tumour subtypes depending on the mesenchymal differentiation involved. Myxoid, osseous, cartilaginous, lipomatous, and xanthomatous subtypes are classified in this group [9]. Tang et al. [10] reported clinicopathological analysis of 15 metaplastic meningiomas, of which 12 tumours were homogenously enhanced and three tumours were heterogeneously enhanced based on contrast MR imaging. The primary location was the dural tissue in approximately 6% of intracranial chondrosarcomas, but radiological examinations principally revealed bone destruction and variable calcification, as well as involvement of neuronal and vascular structures [11].

Calcifications can also develop in chronic subdural haematomas [12]. Dural arteriovenous fistulas with cortical venous reflux may present with calcification in the cortico-medullary junction at the bottom of the cerebral sulci, resulting from chronic venous congestion with impaired perfusion of the involved parenchyma [13]. Cases of idiopathic intracranial dural and optic nerve/sheath calcifications have also been reported [14].

Intracranial physiological calcifications are a common occurrence, found in approximately 35.2% of the adult population [15]. The majority of physiological calcifications appeared in the pineal/habenular region (71%-80%) [15,16], the choroid plexus region (12%-66.2%) [15,16], or the petroclinoid ligament region (8%) [15] or as tentorium cerebella, sagittal sinus, or falx cerebri calcifications (7.3%) [16], vascular calcifications (6.6%) [16], basal ganglia calcifications (0.8%) [16], or lens and other non-defined calcifications (0.9%) [16]. Calcification in the choroid plexus region and the petroclinoid ligament region is typically bilateral [16]. All types of calcifications are increased in older patients, except for lens and other non-defined calcifications. Intracranial physiological calcifications are unaccompanied by any evidence of disease and have no demonstrable pathologic cause. They are often due to calcium or, in certain cases, iron, deposition in the blood vessels of different structures of the brain. CT scan is the most sensitive means of detecting these calcifications [16]. Picht et al. [17] reported a giant intracerebral choroid plexus calcification, measuring approximately 3 × 3 × 4 cm in the right temporal region, which might have had a physiological cause, such as dystrophic calcium salt deposition. In this study, we report on the case of a 47-year-old male patient with a calcified intracranial mass approximately 4.4 × 4.0 × 2.5 cm in volume originating from the transverse sinus and the neighbouring dura. Histological examination demonstrated degenerative alterations, including calcification and ossification, with no evidence of neoplasia or inflammation. The potential mechanism is discussed.

## Case presentation

A 47-year-old man was admitted to our hospital in September 2011 with complaints of intermittent episodes of mild to moderate headache in the right occipital region without nausea or vomiting for two weeks. The neurological examination was normal. The past history revealed hypertension for five years, which was well controlled using oral antihypertensive drugs, and there were no associated systemic complications, such as increased intracranial pressure, intracranial infection or hypercalcaemia.

**Figure 1:**
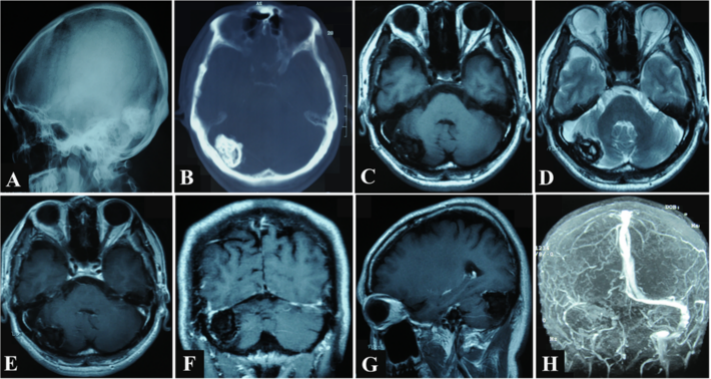
**Preoperative radiological images.** Normal skull X-ray and unenhanced axial computed tomography (CT) scans display an irregular calcified intracranial lesion in the right occipital region, which is separated from the lamina interna cranii by a narrow cleft. Magnetic resonance (MR) images display a blur nodule in the right transverse sinus and the neighbouring dura. The lesion presents with mixed low and equal signal intensity on unenhanced T1-weighted image and mixed low and high signal intensity on unenhanced T2-weighted image. It exhibits irregular linear contrast enhancement only in its dural periphery after administration of gadolinium. Magnetic resonance venogram (MRV) reveals no detectable venous flow of the right transverse sinus and the right sigmoid sinus. **A**, Lateral normal skull X-ray films; **B**, unenhanced axial CT films; **C**, unenhanced axial T1-weighted MR image; **D**, unenhanced axial T2-weighted MR image; **E**, enhanced axial MR image; **F**, enhanced coronal MR image; **G**, enhanced sagittal MR image; **H**, MRV image.

Normal skull X-ray and computed tomography (CT) scans revealed an irregular, calcified, intracranial lesion measuring approximately 4.4 × 4.0 × 2.5 cm in the right occipital region, and the lesion was separated from the lamina interna cranii by a narrow cleft (Figure 1).

Magnetic resonance (MR) examination was performed using a 1.5-T imager. T1- and T2-weighted images were obtained before and after administration of gadopentetate dimeglumine. A blur nodule was delineated in the right transverse sinus and cerebellar tentorium, protruding into the posterior cranial fossa and compressing the right cerebellar hemisphere. The lesion showed mixed low and equal signal intensity on T1-weighted images, and mixed low and high signal intensity on T2-weighted images. The lesion displayed irregular linear contrast enhancement only in its dural periphery. A magnetic resonance venogram (MRV) revealed that there was no detectable venous flow in the right transverse sinus and the right sigmoid sinus (Figure 1).

Normal levels of parathormone, serum calcium and phosphorus excluded diagnoses of hypercalcaemia and hypoparathyroidism.

**Figure 2:**
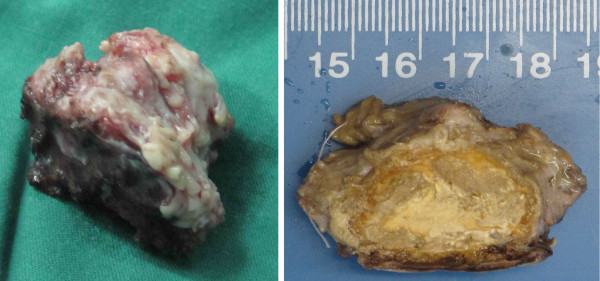
**The calcified and ossified mass after surgical excision.** The mass appeared as irregular, bone-hard, poorly vascularised, and pink and measured approximately 4.4 × 4.0 × 2.5 cm. **Left**, the mass after removal; **right**, the mass after formalin-soaking.

The decision to remove the mass was made based on the suspicion that the mass could be a calcified meningioma. The patient underwent a right occipital craniotomy. There was no adhesion between the skull flap and the dura. A bone-hard, poorly vascularised, pink mass was found, originating from the right transverse sinus and the neighbouring convex dura of the cerebellar hemisphere, as well as the cerebellar tentorium, and tightly adherent to the adjacent cerebellar tissues. Although the MRV images revealed a total occlusion of right transverse sinus, there was still some venous bleeding on the proximal and distal ends of the transverse sinus across the mass, which were first ligated using silk sutures and then cut. After careful dissection around the mass, it was removed totally en bloc with minimal blood loss (Figure 2). The skull flap was replaced.

**Figure 3:**
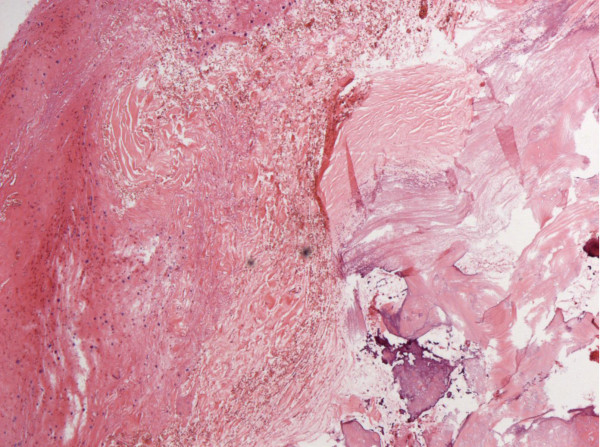
**Haematoxylin- and eosin- stained section (100X).** The image revealed fibrous connective tissue with hyaline degeneration, calcification, and ossification, as well as hyperblastosis of the neighbouring cerebellar glial tissue.

Pathological examination revealed fibrous connective tissue with hyaline degeneration, calcification, and ossification, as well as hyperblastosis of the neighbouring cerebellar glial tissue (Figure 3), with no indication of neoplasia, inflammation or thrombosis of the transverse sinus.

The clinical course after surgery was uneventful. No neurological deficit developed. Following discharge, the patient followed up regularly. His preoperative right occipital headache subsided completely.

**Figure 4:**
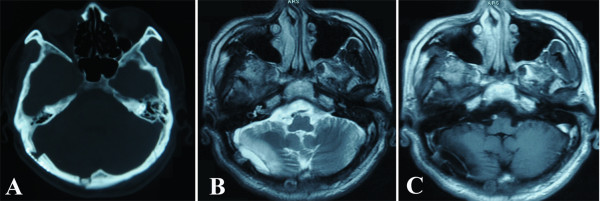
**Follow-up radiological images 17 months after the operation.** These images reveal that the right occipital intracranial mass has disappeared. **A**, unenhanced axial CT image; **B**, unenhanced axial T2-weighted MR image; **C**, enhanced axial MR image.

The latest follow-up radiological images were obtained 17 months postoperatively, and these images showed that the right occipital intracranial mass had disappeared (Figure 4).

The cause of the calcification and ossification of the transverse sinus and neighbouring dura in the present case is not clear. The presence of a calcified meningioma was suspected preoperatively, but the pathologic examination provided no indication of neoplasia or inflammation. Additionally, it did not support calcification of the transverse sinus thrombosis, which characteristically would be located within the sinus [18]. The probable cause might have been physiological degeneration, as the pathologic examination of the mass only revealed degenerative alterations, including the fibrous connective tissue with hyaline degeneration, calcification and ossification. Hyperblastosis of the neighbouring cerebellar glial tissue might have been caused by a response induced by the long-term compression of the mass. As there was total occlusion of right transverse sinus and the patient did not exhibit increased intracranial pressure, the degenerative course should have been long-term enough for the compensation of intracranial venous drainage via the collateral circulation. The identification of the calcified intracranial mass was in fact incidental to the examination pertaining to the patient’s intermittent episodes of mild to moderate headache in the right occipital region, as his headache had only persisted for two weeks. Degenerative calcification and ossification cannot develop in such a short period. The cause of the postoperative disappearance of his headache might have been partially psychological because he no longer had any anxiety regarding the intracranial mass. Nevertheless, if the diagnosis of degenerative calcification and ossification could be considered preoperatively, conservative observation might have been an option. To the best of our knowledge, this is the first description of a case of massive degenerative calcification and ossification originating from the transverse sinus and the neighbouring dura mimicking meningioma.

## Conclusions

We report a rare case of massive calcification and ossification of the transverse sinus and neighbouring dura mimicking meningioma. Degenerative calcification and ossification may serve as a rare differential diagnosis for diseases, such as meningiomas, in the transverse sinus and the neighbouring dura.

## Consent

Written informed consent was obtained from the patient for publication of this case report and any accompanying images. A copy of the written consent is available for review by the Editor-in-Chief of this journal.

## Abbreviations

CT: Computed tomography; MR: Magnetic resonance; MRV: Magnetic resonance venogram.

## Competing interests

The authors declare that they have no competing interests.

## Authors’ contributions

ZX performed the operation, carried out the literature search, and wrote the first and the final versions of the manuscript. CS supervised the diagnosis and treatment, assisted with the operation, and revised the manuscript. YX performed the pathologic diagnosis, provided the pathological figure, and helped to draft the manuscript. All authors read and approved the final manuscript.

## Pre-publication history

The pre-publication history for this paper can be accessed here:

http://www.biomedcentral.com/1471-2377/13/143/prepub

## References

[B1] Al-Motabagani M Haroun H Meguid EA Calcification and ossification of the convexity of the falx cerebri and related subdural space in human cadavers Neurosciences (Riyadh) 2004 9 4 261 264 23377245

[B2] Chen CP Lin SP Lin DS Liu YP Hsu LJ Wang W Clinical imaging findings in a girl with Hutchinson-Gilford progeria syndrome Genet Couns 2012 23 1 1 7 22611635

[B3] Livingston JH Stivaros S van der Knaap MS Crow YJ Recognizable phenotypes associated with intracranial calcification Dev Med Child Neurol 2013 55 1 46 57 10.1111/j.1469-8749.2012.04437.x 23121296

[B4] Elwatidy SM Jamjoom ZA Jamjoom AB Yakoub AO Craniopharyngioma. Analysis of factors that affect the outcome Saudi Med J 2002 23 1 34 38 11938361

[B5] Lee YY Van Tassel P Intracranial oligodendrogliomas: imaging findings in 35 untreated cases AJR Am J Roentgenol 1989 152 2 361 369 10.2214/ajr.152.2.361 2783515

[B6] Kizana E Lee R Young N Dorsch NW Soo YS A review of the radiological features of intracranial meningiomas Australas Radiol 1996 40 4 454 462 10.1111/j.1440-1673.1996.tb00448.x 8996913

[B7] Oya S Kim SH Sade B Lee JH The natural history of intracranial meningiomas J Neurosurg 2011 114 5 1250 1256 21250802 10.3171/2010.12.JNS101623

[B8] Rohringer M Sutherland GR Louw DF Sima AA Incidence and clinicopathological features of meningioma J Neurosurg 1989 71 5 Pt 1 665 672 2809720 10.3171/jns.1989.71.5.0665

[B9] Scheithauer BW Tumors of the meninges: proposed modifications of the world health organization classification Acta Neuropathol 1990 80 4 343 354 10.1007/BF00307686 2239146

[B10] Tang H Sun H Chen H Gong Y Mao Y Xie Q Xie L Zheng M Wang D Zhu H Clinicopathological analysis of metaplastic meningioma: report of 15 cases in huashan hospital Chin J Cancer Res 2013 25 1 112 118 23372349 10.3978/j.issn.1000-9604.2013.01.10 PMC3555304

[B11] Korten AG ter Berg HJ Spincemaille GH van der Laan RT Van de Wel AM Intracranial chondrosarcoma: review of the literature and report of 15 cases J Neurol Neurosurg Psychiatry 1998 65 1 88 92 10.1136/jnnp.65.1.88 9667567 PMC2170168

[B12] Galldiks N Dohmen C Neveling M Fink GR Haupt WF A giant bilateral calcified chronic subdural hematoma Neurocrit Care 2010 12 2 272 273 10.1007/s12028-009-9303-z 19902386

[B13] Metoki T Mugikura S Higano S Ezura M Matsumoto Y Hirayama K Takahashi S Subcortical calcification on CT in Dural arteriovenous fistula with cortical venous reflux AJNR Am J Neuroradiol 2006 27 5 1076 1078 16687546 PMC7975737

[B14] Phadke RV Agarwal P Sharma K Chauhan SS Idiopathic duro-optic calcification–a new entity? Clin Radiol 1996 51 5 359 361 10.1016/S0009-9260(96)80116-4 8641101

[B15] Sedghizadeh PP Nguyen M Enciso R Intracranial physiological calcifications evaluated with cone beam CT Dentomaxillofac Radiol 2012 41 8 675 678 10.1259/dmfr/33077422 22842632 PMC3528191

[B16] Daghighi MH Rezaei V Zarrintan S Pourfathi H Intracranial physiological calcifications in adults on computed tomography in Tabriz, Iran Folia Morphol (Warsz) 2007 66 2 115 119 17594669

[B17] Picht T Stendel R Stoltenburg-Didinger G Brock M Giant intracerebral choroid plexus calcification Acta Neurochir (Wien) 2004 146 11 1259 1261 10.1007/s00701-004-0309-1 15503189

[B18] Davies RP Slavotinek JP James SL Morphett AD Calcified cerebral sinus thrombosis in infancy–CT appearances with pathological correlation Pediatr Radiol 1989 20 1–2 101 103 2602001 10.1007/BF02010649

